# Bringing Psychology Students Closer to People with Schizophrenia at Pandemic Time: A Study of a Distance Anti-stigma Intervention With In-presence Opportunistic Control Group

**DOI:** 10.1007/s40737-022-00308-1

**Published:** 2022-10-06

**Authors:** Lorenza Magliano

**Affiliations:** Department of Psychology, Lorenza Magliano, University of Campania “Luigi Vanvitelli”, Viale Ellittico 31, 81100 Caserta, Italy

**Keywords:** COVID-19, At-distance education, Schizophrenia, Students, Stigma

## Abstract

Psychology students are a target population to increase the likelihood that Persons With Schizophrenia (PWS) will receive evidence-based psycho-social interventions in the future. The willingness of future psychologists to care for PWS can be supported through anti-stigma educational interventions. During the pandemic, university education was delivered largely at-distance, which was later combined with in-presence education. This study explored whether an At-Distance Educational Intervention (ADEI), addressing stigma in schizophrenia via scientific evidence and testimony: would improve psychology students' views of PWS, at the one-month post intervention re-assessments; would be more effective of the same In-Presence Educational Intervention (IPEI). ADEI was delivered online to students of two Master’s degrees in Psychology at the University of Campania “Luigi Vanvitelli”, Caserta, Italy. IPEI was administered to a similar group of 76 students in the pre-pandemic era. Participants completed an anonymous questionnaire about their views on schizophrenia before the intervention (two three-hour sessions one week apart) and one month after its completion. Compared to their pre-intervention assessments, at post-intervention reassessments the 65 ADEI students were: more confident in the recovery and the usefulness of psychological therapies; surer of the PWS awareness and capability to report health problems to professionals; more skeptical about PWS dangerousness, social distance, and affective difficulties; more uncertain on the opportunity to discriminate PWS in hospital and psychology practices. ADEI was more effective than IPEI in five of the ten dimensions analyzed and similarly effective in the remaining others. ADEI may represent a valuable alternative to IPEI for improving future psychologists' view of PWS.

**Supplementary Information:**

The online version contains supplementary material available at 10.1007/s40737-022-00308-1.

## Introduction

Despite the key role of psychologists in the development of evidence-based psychosocial interventions for schizophrenia (McDonagh et al. 2022; Mc Glanaghy et al. 2021; Mueser et al. 2013; Reddy et al. 2010), the involvement of these professionals in the care of persons with this disorder is still limited (O'Connor and Yanos 2021). This is one of the reasons why the availability of non-pharmacological interventions for people with schizophrenia (PWS) remains insufficient in routine clinical settings (Corrao et al. 2022; Ince et al. 2016). Poor involvement of psychologists in schizophrenia can be partially explained by limited training in psychosocial approaches to this disorder in the psychology studies (Buck et al. 2014; Ince et al. 2016; Mojtabai et al. 2011; Roe et al. 2006) and negative attitudes towards PWS among psychologists (Servais & Saunders 2007; Valery & Prouteau 2020). The prevalence of biogenetic causal models of schizophrenia in the psychological field (Deacon 2013) further directs treatment toward an almost exclusively medical-pharmacological pathway (Corrao et al. 2022; Magliano et al. 2017a; Read et al. 2013). Biogenetic models also feed into the view of schizophrenia as an incurable disorder whose symptoms are poorly controllable by the will of the affected person, reinforcing professionals' perception of PWS as dangerous, unpredictable and to be kept at a distance (Harangozo et al. 2014; Read et al. 2013).

Psychology students are a target population to increase the likelihood that PWS will receive integrated evidence-based interventions in the future. Studies reveal that psychology students, similar to students of other health disciplines (LLerena et al. 2002; Magliano et al. 2011; Mannarini et al. 2020; Masedo et al. 2021), sometimes have negative attitudes towards PWS, and that students’ attitudes do not invariably improve during university studies (Economou et al. 2012; Magliano et al. 2013, 2016a; Maranzan 2016). Research shows that it is possible to improve students’ attitudes towards PWS through anti-stigma educational interventions that: include direct or indirect contact with people with this disorder and their involvement as testimonials; frame schizophrenia according to a balanced bio-psycho-social causal model; and refer to a recovery-oriented therapeutic approach (Cangas et al. 2017; Clement et al. 2012; Friedrich et al. 2013; Griffiths et al. 2014; Lincoln et al. 2008; Pingani et al. 2021; Stubbs 2014; Yamaguchi et al. 2013, 2019). In 2011, an anti-stigma educational intervention for health disciplines students, entitled “Social dangerousness and incurability of schizophrenia: prejudices and scientific evidences”, was developed at the University of Campania “Luigi Vanvitelli”, Caserta, Italy (Magliano et al. 2014). When administered to 211 medical and psychology students, the intervention was successful in reducing students’ perception of PWS as dangerous, unpredictable, and affected by an incurable disorder at the immediate follow-up reassessment (Magliano et al. 2014). In a further study at the same University (Magliano et al. 2016b), the intervention was evaluated in a randomly selected sample of 76 psychology students vs. a 112-psychology students’ control group. Compared with their baseline assessment, at one-month post-intervention reassessment, the educated students: endorsed more psychosocial causes and more of them recommended psychologists in the treatment of schizophrenia; were surer about recovery and the usefulness of drug and psychological treatments; and were more skeptical about the unpredictability of PWS. Compared to controls, at one-month re-assessment educated students were more optimistic about recovery and more skeptical regarding unpredictability of PWS.

In the past two years of the pandemic, university learning has been largely based on the usage of distance education, later combined with face-to-face education (Naciri et al. 2021; Dedeilia et al. 2020; Dhawan 2020). Studies examining the effects of at-distance education applied to courses previously delivered in-person found that e-education did not decrease examination scores (Kronenfeld et al. 2021) and it was also well accepted by students (Abdull Mutalib et al. 2022; Dedeilia et al. 2020). Costs and time-saving benefits and difficulties due to unfamiliarity with the e-education approach were reported (Monteduro, 2021; Sharaievska et al. 2022). Few studies examined the capacity of at-distance education to improve future psychologists’ attitudes toward PWS. A controlled study examined the impact of a virtual program to reduce the stigma of severe mental disorders among students of health disciplines (Rodríguez-Rivas et al. 2021). The online program, including project-based learning, clinical simulations with standardized patients and E-Contact with real patients, was found effective in reducing students’ stereotypes, perception of dangerousness, and lack of solidarity toward PWS. A pilot study explored the effects of the Magliano et al. (2014) educational intervention when delivered at-distance (At-Distance Educational Intervention—ADEI) to psychology students (Magliano et al. 2021). Compared to the pre-intervention evaluations, at the one-month follow-up, the participants were: more optimistic about recovery and the usefulness of psychological therapies in schizophrenia; and, more confident in the ability of PWS to behave appropriately when interacting with other people. Given the ongoing pandemic, it would be worth further investigating whether at-distance education improves students’ attitudes toward PWS and whether at-distance education is, in some cases, preferable to in-presence education.

In this study, the effect of ADEI on views of PWS was examined in a sample of 142 psychology students from two master’s degrees at the University of Campania “Luigi Vanvitelli”, Caserta, , Italy, using paired pre-post assessments matched by an anonymous code (primary study aim). Furthermore, the effect of ADEI on students’ attitudes was compared with that of the same intervention delivered in-presence (In-Presence Educational Intervention, IPEI) to the 76-student sample of the Magliano et al. 2016b study (opportunistic control group, secondary study aim).

The study’s primary aim was investigated by measuring the following views in the ADEI sample: 1–2) usefulness of pharmacological and psychological therapies; 3) need of long-term pharmacological therapies; 4) possibility of recovery in schizophrenia; 5) insight of PWS; 6) capacity of PWS to report their health conditions to medical doctors; 7) perception of unpredictability and others’ need for social distance from PWS; 8) perception of dangerousness of PWS; 9) discriminatory behaviors to be adopted with PWS in non-psychiatric hospital wards; 10) difficulties of PWS in having romantic relationships; 11) capacities of PWS to report their mental health problems to psychologists; and, 12) discriminatory behaviors to be adopted with PWS in the psychologist’s office.

It was hypothesized that, compared to their paired baseline assessment, at one-month post intervention re-assessment, ADEI students were: more convinced of the usefulness of pharmacological and psychological treatments and the possibility of recovery in schizophrenia; more skeptical about life-long drugs for the treatment of this disorder; more confident of the awareness of PWS on their own health status and of PWS capacity to report their health problems to physicians and psychologists; more doubtful about the opportunity to adopt discriminatory behaviors against PWS in non-psychiatric hospital wards and in the psychologists' offices; and, more skeptical regarding the dangerousness of, and social distance from, PWS and the difficulties these persons experience in emotional relationships.

As the study secondary aim, it was hypothesized that ADEI would be more effective than IPEI to improve psychology students’ views of schizophrenia at the one-month re-assessment. ADEI and IPEI groups were compared in all the above-mentioned views, except views n. 11 and n. 12 (not collected in the IPEI sample). The IPEI data used in this study had not been analyzed previously, except for six single items included in the 2–3 and 7–8 subscales views listed above.

## Methods

### Study Design

The ADEI was scheduled within the laboratory activities included in the Psychiatry course for psychology students of the master’s degrees in "Clinical Psychology" (2nd semester of the 1st year) and in "Applied Psychology" (1st semester of the 2nd year) at the University of Campania “Luigi Vanvitelli”, Caserta, Italy. Before the start of the ADEI, students (expected participants: N = 99 per course) were invited to participate in an online voluntary evaluation of their views of mental disorders to be repeated one month after the ADEI completion. Students who accepted were asked to complete online the Opinions on mental illness Questionnaire—revised version (OQ; Magliano et al. 2017b), after reading an ICD-10 clinical description of schizophrenia. Students were also asked to report on the OQ an anonymous code to be used for matching their baseline and one-month post-intervention re-assessments. Participants completed the OQ online via their own digital devices, using a link posted on the Microsoft Teams channel dedicated to the psychiatry course. Answers were collected using the Google forms app. The possibility to stop the completion of the questionnaire and delete the answers was guaranteed by the online mode (just closing the Internet page without saving). The ADEI was delivered in October–November 2020 and in March–May 2021. The IPEI control group had received the intervention in March–April 2012 and March–April 2013 (see Magliano et al. 2016b, for further details). The study was approved by the ethics committee of the Department of Psychology of the University of Campania “Luigi Vanvitelli”, Caserta, Italy, approval released to the IPEI study protocol, being only changed the intervention delivery mode.

## Contents of the Educational Intervention

The educational intervention consisted of two three-hour sessions with an interval of a week between them. The first session covered the following topics: a) definitions of stigma; b–c) research studies and personal stories on stigma and its effects; d) stigma and the media; e) stigma and mental health problems; f) stigma against persons with mental disorders in health contexts; g) stigma in schizophrenia. The second session focused on: a–c) scientific evidence on dangerousness in “at risk” minority social groups, persons with mental disorders, and particularly schizophrenia; d) subjective and objective dimensions of recovery; e) evidences on recovery in schizophrenia; and f) empowerment-oriented mental health services. Scientific reports, media articles, and video materials from anti-stigma campaigns were used in both sessions. In addition, four people who have been recovered or experienced stigma related to their mental health problems provided audio testimonies of their personal stories. ADEI was delivered on Microsoft Teams platform. Participants were prompted to intervene in plenary discussions, verbally, by using the audio link with or without video, or in writing on the chat.

## Assessment Instrument

The OQ included: a) 16 yes/no items exploring factors involved in the development of schizophrenia; b) 4 yes/no items about which professionals should be involved in the care of PWS; c) 23 items grouped into 10 subscales and addressing respondent’s views on: 1–2) usefulness of pharmacological and psychological therapies; 3) need of long-term pharmacological therapies; 4) possibility of recovery; 5) insight of PWS; 6) capacity of PWS to report their health conditions to medical doctors; 7) perception of social distance from PWS; 8) perception of dangerousness; 9) discriminatory behaviors to be adopted with PWS in non-psychiatric hospital wards; and 10) difficulties of PWS in having romantic relationships. Section c items are rated on a 3-point scale, from 1 = “not true” to 3 = “completely true”. The psychometric properties of OQ section C, were preliminary tested and found to be satisfactory (Confirmatory Factor Analysis: model χ^2^ = 320.35, df 188, N = 387, *p* < 0.05; NNFI = 0.95; CFI = 0.97; RMSEA = 0.04 (0.03; 0.05); SRMR = 0.04; all factor loadings significant for *p* < 0.001; Cronbach’s alpha values of the factors ranging from 0.65 to 0.83; Magliano et al. 2017b). For the purposes of this study, a further 5 items were added to examine respondent views of: 11) capacities of PWS to report their mental health problems to psychologists; and 12) discriminatory behaviors to be adopted with PWS in psychologist’ office. In this study sample, Cronbach’s alpha values computed on OQ section c, and two additional subscales ranged from 0.61 to 0.94 (OQ a–b sections not analysed).

## Statistical Analysis

Descriptive statistics were computed on OQ single items and participants’ demographic characteristics (age and gender). Pre-post data were matched using the anonymous code that students were asked to report on the questionnaire. In the ADEI group, General Linear Model (GLM) for repeated measures was computed to explore differences in pre vs. one-month post-intervention mean scores of the OQ section c and additional subscales (within subject factor: pre-post paired assessment). The same analysis was used to compare pre-post mean score of the 10 OQ section c factors in the ADEI vs. IPEI group (within subject factor: pre post paired assessment; between subject: type of education: ADEI vs. IPEI). GLM analyses were adjusted for age and gender, as appropriate. The statistical significance level was set at *p* < 0.05. Analyses were performed using Statistical Package for the Social Sciences (SPSS) Version 21.

## Results

### Flow and Socio-demographic Characteristics of ADEI and IPEI Participants

**Fig. 1:**
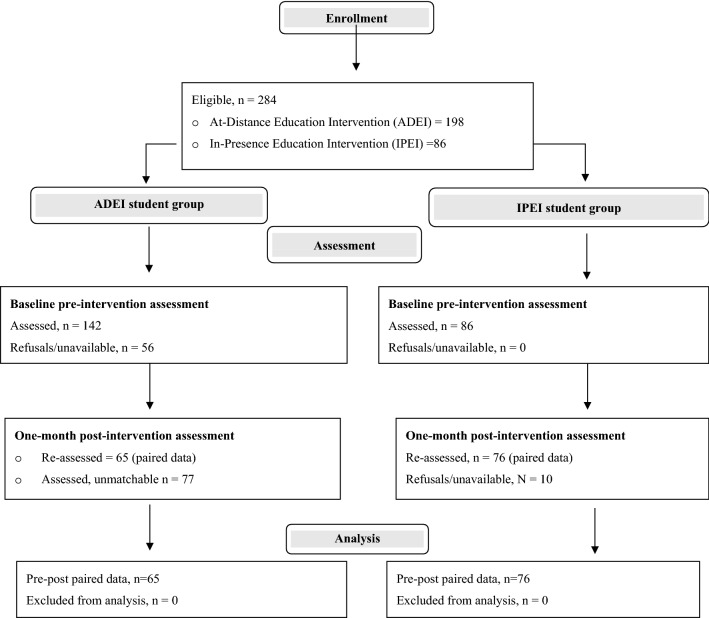
Participant Flow Diagram

As shown in the flowchart (Fig. 1), 142 out of the 198 expected ADEI participants completed the OQ at baseline and 142 participants completed the same questionnaire at the one-month post intervention (participation rate: 71.7%; 131, 92.3% female, average age 24.1 ± 3.5 (sd) years). Sixty-five participants provided a matchable code and 77 did not (40 “can’t remember the code”, 37 blank/non matchable, attrition rate: 54.2%). Of the 65 students providing a matchable code, 63 (96.9%) were female and 2 (3.1%) were male, and they had and average age of 24.2 ± 3.3 (sd) years. Non-matchable students were similar to matchable ones for sex and age. The IPEI control group included 76 students of the Applied Psychology Master’s degree who completed the paired pre- and one-month post-intervention assessment (attrition rate: 11.6%) and provided an anonymous matchable code. Of these, 66 (86.8%) were female and 10 (13.2%) were male, and they had and average age of 26.9.2 ± 5.8 (sd) years. Of the 141 students with paired pre-post matchable assessments (ADEI 65 and IPEI 76), 91.5% were female and they had an average age of 25.7 ± 4.9 (sd) years. Compared to the 65 ADEI students, the 76 IPEI students were slightly more frequently male (2 vs. 10, χ^2^ 4.6, df 1, *p* < 0.03, Yates’ correction for continuity: 3.4, df 1, *p* = 0.07), and they had a higher average age (*F* = 10.7, df 1, 138, *p* < 0.001). As the distribution of the age variable was positively skewed (skewness = 3.7, ex 0.20; kurtosis = 165.0, ex 0.20), in the multivariate analyses normalized age score was used. Furthermore, given the very low number of male students (2 in ADEI and 10 in IPEI), GLM was only adjusted for normalized age score.

## Views of Schizophrenia in ADEI Students: Pre-intervention Descriptive Results

Of the 142 ADEI students who completed their pre-intervention assessment, 22.9% firmly stated that PWS could recover, 25.2% believed that these persons had to take drugs life-long, and 38.0% were convinced that psychological treatments were useful in schizophrenia (complete data in Supplementary File 1, additional Table 1). Seventy-three percent of respondents felt that PWS were kept at a distance, and 16.9% perceived these persons as being dangerous to others. Twelve percent of students thought that PWS should be separated from other patients in non-psychiatric hospital wards and 19.5% believed that PWS should be accompanied when going to psychology offices.

## Views of Schizophrenia in ADEI Students: Paired Comparisons of Pre-intervention Versus One-month Post-intervention Assessments

The GLM performed on the paired ADEI data (*N* = 65) is shown in Table 1. GLM revealed a significant effect of the intervention on all OQ section c and two additional subscales (Wilks’ *λ* = 0.25, *F* (overall df 12, 52) = 12.7, *p* < 0.0001). The effect of the covariate age was not significant (age: Wilks’ *λ* = 0.74, *F* (overall df 12, 52) = 1.5, *p* = 0.15; age x intervention: Wilks’ *λ* = 0.83, *F* (overall df 12, 52) = 0.90, *p* = 0.55). *C*ompared to their pre-intervention assessment, at post-intervention students were surer that PWS could recover and that psychological therapies were useful in schizophrenia. Moreover, students were more skeptical about the need of life-long drug therapies in schizophrenia. At one-month reassessment, students were surer that PWS were aware of their disorder and capable to report their problems to professionals. Students were also less convinced that PWS were dangerous and kept at a distance and that they had difficulties in romantic relationships. Finally, at post interventions students were more uncertain on the opportunity to discriminate PWS in non-psychiatric wards and psychology offices.

**Table 1:** Psychology Students’ Views of Schizophrenia: Paired Comparisons of Baseline Versus One-Month Reassessment in the ADEI group (N = 65)

Subscales	Within subject factor: baseline and one-month reassessment		
	Mean ± se		F* _(1, 63)_
	Baseline	One-month reassessment	
Possibility to recover	2.2 ± .06	2.9 ± .05	96.1^b^
Usefulness of drug therapies	2.4 ± .07	2.6 ± .06	3.3
Usefulness of psychological therapies	2.6 ± .06	2.9 ± .04	9.8^a^
Need of long-term drug therapies	1.8 ± .05	1.4 ± .04	29.9^b^
Insight of PWS	1.8 ± .06	1.4 ± .05	9.2^a^
Perception of social distance from PWS	2.5 ± .05	2.2 ± .05	27.3^b^
Perception of dangerousness	1.9 ± .04	1.6 ± .05	33.1^b^
Treatments of PWS in non-psychiatric hospital wards	1.8 ± .05	1.3 ± .05	52.0^b^
Reliability of PWS in reporting their health condition to MDs	1.8 ± .05	2.4 ± .05	39.4^b^
Difficulties of PWS in having romantic relationships	2.1 ± .07	1.5 ± .07	26.7^b^
Reliability of PWS in reporting their health condition to psychologists	1.8 ± .06	2.4 ± .06	35.2^b^
Treatments of PWS in psychologist office	1.5 ± .04	1.1 ± .02	51.1^b^

*ADEI* At-distance Eeducational Iintervention; *PWS* Persons With Schizophrenia;

^a^*p* < 0.005; ^b^*p* < 0.0001;

^*^F values refer to individual comparisons

Analysis adjusted for age (normalized score);

## Pre-post Paired Comparisons of Views of Schizophrenia in ADEI Versus IPEI Student Groups

As reported in Table 2, GLM performed on the 65 ADEI vs. the 76 IPEI students revealed statistical significant effects on students' views of the intervention (pre-post assessments: Wilks’ *λ* = 0.30, *F* (overall df 10, 129) = 29.8, *p* < 0.0001), the delivery mode (ADEI vs. IPEI: Wilks’ *λ* = 0.64, *F* (overall df 10, 129) = 7.1, *p* < 0.0001) and the interaction between them (Wilks’ *λ* = 0.79, *F* (overall df 10, 129) = 3.41, *p* < 0.001). The effect of the covariate was not significant (age: Wilks’ *λ* = 0.91, *F* (overall df 10, 129) = 1.26, *p* = 0.26; age x intervention: Wilks’ *λ* = 0.96, *F* (overall df 10, 129) = 0.50, *p* = 0.88). The interaction between delivery mode and the intervention revealed changes in favor of the ADEI group as far as: students’ views of recovery in schizophrenia; reliability of PWS in reporting their disorders to MDs; and, dangerousness, social distance and discrimination of PWS in non-psychiatric hospital wards.

**Table 2:** Psychology Students’ Views of Schizophrenia: Paired comparisons of baseline versus one-month reassessment in the ADEI (N = 65) vs. IPEI groups (N = 76)

Subscales	Within subject factor: Baseline and one-month reassessment			Between-subject factor: Education type			Interaction effects: Baseline and one-month reassessment x education type				
	Mean ± se			Mean ± se			ADEI, Mean ± se		IPEI, Mean ± se		
	Baseline	One-month reassessment	F* _(1, 138)_	ADEI	IPEI	F* _(1, 138)_	Baseline	One-month reassessment	Baseline	One-month reassessment	F* _(1, 138)_
Possibility to recover	2.2 ± 0.04	2.8 ± 0.03	129.0^d^	2.6 ± 0.04	2.4 ± 0.04	13.7^d^	2.2 ± 0.06	2.9 ± 0.05	2.2 ± 0.05	2.6 ± 0.05	7.1^b^
Usefulness of drug therapies	2.3 ± 0.05	2.5 ± 0.04	21.9^d^	2.5 ± 0.05	2.4 ± 0.05	1.1	2.4 ± 0.07	2.6 ± 0.06	2.3 ± 0.06	2.5 ± 0.06	0.58
Usefulness of psychological therapies	2.6 ± 0.04	2.8 ± 0.03	35.5^d^	2.7 ± 0.04	2.7 ± 0.04	0.18	2.6 ± 0.06	2.9 ± 0.04	2.6 ± 0.06	2.8 ± 0.04	0.07
Need of long-term drug therapies	1.8 ± 0.03	1.5 ± 0.03	89.6^d^	1.6 ± 0.04	1.7 ± 0.03	3.5	1.8 ± 0.05	1.4 ± 0.04	1.9 ± 0.04	1.5 ± 0.04	0.31
Insight of PWS	1.9 ± 0.04	1.6 ± 0.03	25.9^d^	1.6 ± 0.04	2.0 ± 0.04	47.3^d^	1.8 ± 0.06	1.4 ± 0.05	2.1 ± 0.06	1.9 ± 0.05	3.3
Perception of social distance from PWS	2.5 ± 0.03	2.3 ± 0.03	23.4^d^	2.4 ± 0.04	2.4 ± 0.04	0.12	2.5 ± 0.05	2.2 ± 0.05	2.4 ± 0.05	2.3 ± 0.04	7.8^b^
Perception of dangerousness	2.0 ± 0.03	1.7 ± 0.03	61.4^d^	1.7 ± 0.04	1.9 ± 0.03	10.8^c^	1.9 ± 0.04	1.6 ± 0.05	2.0 ± 0.04	1.8 ± 0.04	4.5^a^
Treatments of PWS in non-psychiatric hospital wards	1.9 ± 0.03	1.4 ± 0.03	105.5^d^	1.6 ± 0.04	1.7 ± 0.04	12.7^c^	1.8 ± 0.05	1.3 ± 0.05	1.9 ± 0.05	1.6 ± 0.04	7.2^b^
Reliability of PWS in reporting their health condition to MDs	1.8 ± 0.03	2.2 ± 0.03	75.8^d^	2.1 ± 0.03	2.0 ± 0.03	5.8^a^	1.8 ± 0.05	2.4 ± 0.05	1.9 ± 0.05	2.1 ± 0.04	19.7^d^
Difficulties of PWS in having romantic relationships	2.1 ± 0.05	1.5 ± 0.04	73.6^d^	1.8 ± 0.05	1.8 ± 0.05	0.17	2.1 ± 0.07	1.5 ± 0.07	2.0 ± 0.07	1.5 ± 0.06	0.37

*ADEI* At-Distance Educational Intervention; *IPEI* In-Presence Educational Iintervention; *PWS* Persons With Schizophrenia;

^a^*p* < 0.05; ^b^*p* < 0.01; ^c^*p* < 0.001; ^d^*p* < 0.000;

^*^F values refer to individual comparisons

Analysis adjusted for age (normalized score);

## Discussion

### Critical Comments of the Data

The results of this study confirm that ADEI is able to change students' views on PWS, which may influence the relationship between psychologists and PWS in the future. Furthermore, the study revealed a superiority of the ADEI over the IPEI in five of the ten analyzed dimensions and a substantial equality of effect in the remaining ones. These encouraging results may be partially related to the characteristics of the intervention, as it was based on indirect contact with people who recounted their experience of mental disorders, emphasizing the support received from families and friends and the health professionals in the recovery process (Soundy et al. 2015). Recounting these aspects may have contributed to participants’ increased acknowledgement of the usefulness of psychological therapies and the role of psychologists in the care of the disorder. For instance, a user highlighted the importance of psychotherapy in her recovery process as well as “the luck” of having a “human” relationship with mental health professionals (Tibaldi & Govers 2012). She also outlined the beneficial effects of antipsychotics on symptoms without denying the side effects of the long-term use of such drugs (Magliano et al. 2016b).

At one-month follow up reassessment, students were more confident regarding recovery in schizophrenia. This result is likely related to testimonies confirming that recovery is possible and the use of literature findings on favorable outcomes of schizophrenia, especially when biopsychosocial treatments are provided (Huxley et al. 2021; Levine et al. 2010; Tibaldi & Govers 2012). Emphasizing the usefulness of psychological therapies in the recovery process is of great importance in the management of schizophrenia, as it may influence future psychologists’ willingness to work with clients with this disorder (Magliano et al. 2016a; Servais & Saunders 2007). At the one month follow up re-assessment, participants were more skeptical regarding the dangerousness and social distance of PWS and the need to discriminate PWS in hospital and psychology practice. These views might have been influenced by discussing experiences and research revealing that the risk of aggressive behaviors is modest in PWS, particularly when these persons receive appropriate treatments and are not in an acute psychotic episode (Fazel et al. 2009; Large et al. 2009). Change in perceived dangerousness might have had an indirect impact on students’ conviction about the opportunity to treat these persons differently from the others in health contexts (Corrigan et al. 2002; Magliano et al. 2017c). Furthermore, the fact that the educational intervention addressed stigma from a social perspective, inscribed PWS’ difficulties into those experienced by other stigmatized groups (stigma against people with developmental disabilities, and against LGBT + , nomad and migrant people), thus avoiding fueling 'a stigma within stigma'. At follow-up re-assessment, students tend to give PWS more credit for awareness of their disorders and the ability to discuss their health problems with doctors and psychologists. This is probably related to testimonies showing that having a mental disorder is not a pervasive experience that invalidates the critical capabilities of the individual as a whole, e.g., by demonstrating that one is able to present one's story as that of people with problems rather than patients.

In terms of the way the educational intervention is delivered, the extensive use of audio and video materials makes it particularly suitable for online use. In the ADEI, participants were prompted to intervene in plenary discussions via chat or using audio only. These options may have facilitated the participation of those shyest students who would have felt uncomfortable intervening in person in the classroom. It should also be considered that ADEI students were likely more attentive due to less tiredness, due to no travel time and distraction by classmates (Dedeilia et al. 2020; Naciri et al. 2021).

## Strengths and Weaknesses of the Study

This is the first study on the effects of an ADEI on psychology students' views of PWS in Italy. Among the strengths of the study, there are: having considered the impact of the intervention on a range of opinions covering respondents’ views of schizophrenia and the relationship of future psychologists with PWS. These opinions were collected online using a validated questionnaire, which may facilitate the replication of the study in other students’ contexts; the use of an opportunistic control group of students who had received the same intervention in attendance. This allows any differences between the groups to be attributed primarily to the delivery mode, contributing to clarify whether at-distance education can be valued as an alternative to in-presence education; the implementation of the ADEI after the first six months of the pandemic, so as to assess the effectiveness of the intervention on learning net of the effect of students’ initial adaptation to remote education. The study has a number of limitations suggesting caution in the interpretation of its results, such as: the sample including predominantly female students (2 male students in ADEI and 10 male students in IPEI), a situation that reflects the high number of female students attending psychology degree courses in Italy (Consorzio Alma Laurea, 2022) and makes the data non-generalizable to male students; the opportunistic control group that had received IPEI about eight years earlier. Within eight years, the content of some psychology courses may have changed in favor of a more biogenetically oriented model of schizophrenia (Deacon, 2013) and this may have influenced participants' interest in PWS. Furthermore, the one-month follow-up reassessment does not allow to understand if changes are maintained over time. Another weakness is the high number of non-matchable questionnaires in the ADEI group, which may have increased the magnitude of differences between groups. Indeed at post-intervention, non-matchable ADEI students showed higher level of social distance (2.2 ± 0.4 vs. 2.4 ± 0.3, *F* = 4.3, *p* < 0.04), perceived dangerousness (1.6 ± 0.4 vs. 1.7 ± 0.4, *F* = 7.3, *p* < 0.008) and prognostic pessimism (2.9 ± 0.2 vs. 2.8 ± 0.4, *F* = 10.4, *p* < 0.002; MANOVA, Wilkins’s λ 0.84, F = 2.06, df 12, 129, *p* < 0.02) than matchable students. However, the comparison of the 142 ADEI students (matchable + non-matchable participants) with the 76 IDEI students at post-intervention re-assessment confirmed statistical significant differences in favor of ADEI, Wilkins’s λ 0.87, F = 2,96, df 10, 207, p < 0.002) in the subscales’ mean scores of insight (1.4 ± 0.5 vs. 1.6 ± 0.4, *F* = 7.8, df1, *p* < 0.006), reliability (2.3 ± 0.5 vs. 2.1 ± 0.3, *F* = 10.9, df1, *p* < 0.001) and dangerousness of PWS (1.6 ± 0.4 vs. 1.8 ± 0.4, *F* = 7.6, df1, *p* < 0.006), and recovery (2.8 ± 0.3 vs. 2.6 ± 0.5, *F* = 13.9, df1, *p* < 0.0001). These results support the evidence of the superiority of ADEI vs. IPEI, even considering the attrition rate. Finally, it should be considered that, as most research on anti-stigma interventions, this study evaluated attitudes and not behaviors towards PWS. To be able to measure the practical effects of such interventions, research is needed to ascertain whether psychologists exposed to anti-stigma interventions during their university career are more often engaged in therapeutic relationships with PWS.

## Conclusions

Despite the weaknesses listed above, this study suggests that ADEI may be useful in improving future psychologists' views of PWS, and that ADEI is a valuable alternative to IPEI. More generally, the results of this study support the potential usefulness of at-distance education in university training for future psychologists. The foreseeable reduction in training costs and the possibility of extending the student population to include those unable to attend in person, suggest the advisability of investing more in this didactive modality in terms of digital infrastructure and university staff training. Such investments are even more necessary given the subsequent waves of pandemics and the likely need to maintain hybrid education, also as a strategy of contagion containment.

## Supplementary Information

Below is the link to the electronic supplementary material.

Supplementary file1 (DOCX 19 KB)

## Data Availability

The data that support the findings of this study are available from the corresponding author upon reasonable request, which must include a protocol and statistical analysis plan and not be in conflict with her publication plan. Declaration.
