# Supplementary material for: Bringing Psychology Students Closer to People with Schizophrenia at Pandemic Time: A Study of a Distance Anti-stigma Intervention With In-presence Opportunistic Control Group
Source: J Psychosoc Rehabil Ment Health. 2022 Oct 6:1–13. Online ahead of print. doi: 10.1007/s40737-022-00308-1 (PMC9534736; doi:10.1007/s40737-022-00308-1)
Supplement: Supplementary file 1 — Supplementary file1 (DOCX 19 KB) [file 40737_2022_308_MOESM1_ESM.docx]

| **Additional Table 1. Views of schizophrenia among the ADEI psychology students: baseline and one-month follow up re-assessment (N=142).** | | | | | | |
| --- | --- | --- | --- | --- | --- | --- |
|  | Baseline | | | One-month follow up | | |
| Items | Not true | Partially true | Completely true | Not true | Partially true | Completely true |
|  | N (%) | N (%) | N (%) | N (%) | N (%) | N (%) |
| § can recover | 1 (0.7) | 107 (76.4) | 32 (22.9) | 0 | 21 (14.8) | 121 (85.2) |
| Drugs are useful for § | 3 (2.2) | 82 (59.9) | 52 (38.0) | 1 (0.7) | 54 (38.0) | 87 (61.3) |
| Psychological interventions are useful for § | 0 | 56 (39.7) | 85 (60.3) | 0 | 18 (12.7) | 124 (87.3) |
| § must take drugs over the life | 41(34.5) | 48 (40.3) | 30 (25.2) | 78 (55.7) | 57 (40.7) | 5 (3.6) |
| If stop taking drugs, § become dangerous | 55(44.7) | 66 (53.7) | 2 (1.6) | 112 (83.0) | 22 (16.3) | 1 (0.7) |
| If stop taking drugs, § become unwell again | 16 (12.8) | 91 (72.8) | 18 (14.4) | 46 (33.8) | 82 (60.3) | 8 (5.9) |
| § do not realize that they are ill | 39 (29.3) | 80 (60.2) | 14 (10.5) | 89 (63.3) | 45 (32.1) | 6 (4.3) |
| § do not realize when they become unwell | 56 (45.5) | 55 (44.7) | 12 (9.8) | 85 (60.3) | 50 (35.5) | 6 (4.3) |
| § are unpredictable | 12 (9.0) | 91(67.9) | 31 (23.1) | 76 (54.3) | 58 (41.4) | 6 (4.3) |
| § are kept at distance by the others | 15(10.8) | 62 (44.6) | 62 (44.6) | 14 (9.9) | 52 (36.6) | 76 (53.5) |
| People does not know how to behave with § | 3 (2.1) | 35 (24.6) | 104 (73.2) | 1 (0.7) | 61 (43.3) | 79 (56.0) |
| People does not understand the difficulties experienced by § | 1 (0.7) | 49 (34.5) | 92 (64.8) | 3 (2.1) | 59 (41.5) | 80 (56.3) |
| People is frightened by § | 3 (2.1) | 52 (36.6) | 87 (61.3) | 3 2.1() | 49 (34.5) | 90 (63.4) |
| §are dangerous to themselves | 7 (5.1) | 106 (77.9) | 23 (16.9) | 24 (17.3) | 103 (74.1) | 12 (8.6) |
| §are dangerous to others | 30 (23.1) | 92 (70.8) | 8 (6.2) | 84 (60.4) | 54 (38.8) | 1 (0.7) |
| In a non-psychiatric hospital ward, §create discomfort to other patients | 43 (42.6) | 49 (48.5) | 9 (8.9) | 106 (76.8) | 25 (18.1) | 7 (5.1) |
| In a non-psychiatric hospital ward, §should be separated from other patients | 61 (59.8) | 29 (28.4) | 12 (11.8) | 113 (83.1) | 20 (14.7) | 3 (2.2) |
| When § are admitted to non-psychiatric wards, psychiatric advice should always be requested | 17 (15.5) | 33 (30.0) | 60 (54.5) | 63 (48.5) | 41 (31.5) | 26 (20.0) |
| In non-psychiatric hospital wards, §should be supervised (for instance, by additional nurse) | 26(27.1) | 51 (53.1) | 19 (19.8) | 98 (74.8) | 31 (23.7) | 2 (1.5) |
| § are reliable in referring their mental problems to medical doctors | 40 (33.3) | 75 (62.5) | 5 (4.2) | 11 (8.1) | 83 (61.5) | 41 (30.4) |
| § are reliable in referring their physical problems to medical doctors | 24(20.9) | 83 (72.2) | 8 (7.0) | 8 (5.9) | 72 (52.9) | 56 (41.2) |
| It is difficult for § to have a love relationship | 20(17.1) | 65 (55.6) | 32 (27.4) | 61 (44.2) | 66 (47.8) | 11 (8.0) |
| It is difficult for § to get married or to live together with a partner | 18 (15.7) | 66 (57.4) | 31 (27.0) | 65 (46.8) | 64 (46.0) | 10 (7.2) |
| § are reliable in referring their mental problems to psychologists | 21 (17.4) | 94 (77.7) | 6 (5.0) | 6 (4.3) | 81 (58.3) | 52 (37.4) |
| In a psychology office, §create discomfort to other clients | 62 (57.4) | 43 (39.8) | 3 (2.8) | 122 (90.4) | 11 (8.1) | 2 (1.5) |
| In a psychology office, §should be separated from other patients | 96 (85.7) | 14 (12.5) | 2 (1.8) | 135 (97.8) | 3 (2.2) | 0 |
| In a psychology office wards, §should be accompanied (for instance, by a relative) | 42 (34.1) | 57 (46.3) | 24 (19.5) | 87 (63.5) | 46 (33.6) | 4 (2.9) |
| In non-psychiatric hospital wards, §should be supervised (for instance, by additional nurse) | 83 (76.1) | 25 (22.9) | 1 (0.9) | 130 (94.9) | 7 (5.1) | 0 |

ADEI=At-Distance Educational Intervention; §=Persons With Schizophrenia (PWS).
